# A Multidimensional Data Set for Obsessive‐Compulsive Disorder to Strengthen Research and Care: A Cross‐Sectional Delphi Study

**DOI:** 10.1002/hsr2.72861

**Published:** 2026-08-13

**Authors:** Farzaneh Asadilari, Ahmad Naghibzadeh‐Tahami, Sadrieh Hajesmaeel‐Gohari, Mohammad Mehdi Ghaemi

**Affiliations:** ^1^ Faculty of Management and Medical Information Sciences, Kerman University of Medical Sciences Kerman Iran; ^2^ Health Foresight and Innovation Research Center, Institute for Futures Studies in Health Kerman University of Medical Sciences Kerman Iran; ^3^ Medical Informatics Research Center, Institute for Futures Studies in Health Kerman University of Medical Sciences Kerman Iran; ^4^ Department of Health Information Sciences, School of Management and Medical Information Sciences, Head of Medical Informatics Research Center, Institute for Futures Studies in Health Kerman University of Medical Sciences Kerman Iran

**Keywords:** MDS, minimum data set, obsessive‐compulsive disorder, OCD

## Abstract

**Background and Aims:**

Obsessive‐compulsive disorder (OCD) is a complex psychiatric condition marked by persistent obsessions and compulsions, leading to significant disability and reduced quality of life. Despite effective treatments, delays in diagnosis and fragmented data collection hinder optimal care. Current assessment tools focus primarily on symptoms and severity, lacking comprehensive data on demographics, environmental factors, and service utilization. This study aimed to develop a multidimensional minimum data set (MDS) for OCD to standardize data collection, improve clinical decision‐making, and support epidemiological research.

**Methods:**

A modified Delphi technique was used, engaging 20 experts (psychiatrists, psychologists, epidemiologists, and health information management specialists) across two consensus rounds. An initial 94 data elements were evaluated, and the final MDS was categorized into seven domains.

**Results:**

In the first Delphi round, experts immediately approved 39 items and rejected 4, while 51 required further evaluation. The second round yielded consensus on 27 additional items. The final MDS comprised 66 essential elements, with 28 items ultimately excluded from the final dataset.

**Conclusion:**

The proposed MDS bridges gaps in existing OCD tools (e.g., Y‐BOCS, DOCS) by incorporating multidimensional data. Its implementation could enhance personalized care, inform public health policies, and address disparities in low‐resource settings. Future validation studies are needed to assess its impact on patient outcomes and global applicability.

## Summary

- A multidimensional MDS for OCD was developed using a modified Delphi technique.
- 66 out of 94 data elements achieved consensus after two rounds.
- The MDS includes seven domains covering clinical, demographic, environmental, and service use data.
- This tool complements existing OCD symptom scales for research and clinical care.

## 1 Introduction

Obsessive‐compulsive disorder (OCD) is a highly complex psychiatric disorder, posing significant challenges in treatment and often resulting in functional and occupational impairments, as well as reduced quality of life. Without proper treatment, OCD can cause severe distress and increase mortality risk [1]. Effective treatment requires integrated approaches that address OCD's complexity to improve outcomes and mitigate the potentially devastating consequences associated with this disorder [2].

OCD is often comorbid with a variety of other psychiatric disorders including psychotic disorders, anxiety disorders, major depressive disorder (MDD), substance use disorder, and bipolar disorder (BD) [3]. These overlapping conditions obscure diagnosis and complicate treatment, emphasizing the need for more extensive research into this condition [4].

The American Psychiatric Association's Diagnostic and Statistical Manual of Mental Disorders (DSM) fifth edition, classified OCD as a condition characterized by the presence of both obsessions (recurrent, irrational, and intrusive thoughts or urges) and compulsions (repetitive behaviors) that significantly impact a person's quality of life [5]. According to the World Health Organization (WHO, 1999), OCD is ranked among the top 10 leading causes of disability worldwide and has been linked to an increased risk of early mortality [6].

Epidemiological studies generally indicate that OCD affects 0.25%–4% of the population [7] with an even larger proportion of individuals experiencing sub‐threshold symptoms [8] and many choosing to hide their disorder from others [8] and prevalence estimates vary due to the specific (DSM) criteria employed to establish the diagnosis [9] and the scarcity of data on mental health in low‐ and middle‐income countries [10].

Although obsessive‐compulsive and related disorders often have a considerable impact on individuals' lives, frequently go underdiagnosed and undertreated. On average, it takes approximately 4.0 years (with a mean of 7.96 years) for individuals with OCD to receive a correct diagnosis and begin appropriate treatment, from the time they first start experiencing symptoms of the disorder [11] Despite the availability of effective treatments for OCD, delay in seeking help is also common, and some individuals may never pursue treatment [12]. Barriers include clinician/patient unawareness, symptom‐related shame, lack of insight, and family members accommodating compulsive behaviors, can contribute to these delays [9]. Without proper treatment, the symptoms of OCD can fluctuate over time but tend to persist and follow a long‐term course [13].

The scarcity of data on adolescent mental health in low‐ and middle‐income countries is a significant concern. This lack of data is compounded by the limited availability of brief instruments for measuring adolescent mental health that have been validated for use in these contexts, making it difficult to accurately assess and monitor mental health at a population level [10].

Advancing OCD epidemiology necessitates a global consensus on a standardized assessment protocol, involving the creation and validation of culturally sensitive research tools. Conducting studies to examine sociodemographic, clinical, and prognostic factors across nations will contribute to better identification, diagnosis, and treatment of OCD worldwide [14]. Accurately measuring mental health across diverse settings necessitates the use of dependable measurement tools. At a population level, this involves the employment of mental health scales that assess individuals' symptoms [10].

Designing a multidimensional minimum data set (MDS) as a set of standard data elements is the initial step in collecting and managing data and information of disease [15]. Implementation of an MDS facilitates collaboration between care providers and fosters data‐driven decision‐making processes [16]. MDS generates valuable statistical data and reports, catering to the needs of care providers and financial stakeholders, and improving healthcare quality, efficiency, and cost‐effectiveness [17]. Furthermore, it guides policymakers, software experts, programmers, and health data managers on essential data elements for system design, ensuring standardized information collection and leading to the development of effective data management systems that improve healthcare decision‐making, policy development, and quality of care [18]. Implementing this tool would also assist epidemiologists in accurately determining OCD prevalence and characterizing its various aspects across different regions.

To the best of our knowledge, there is currently no established MDS specifically designed to collect information on OCD. Developing such an MDS would contribute to advancing scientific understanding and accumulating valuable data on the disorder. The strict adoption and use of an OCD‐specific MDS, particularly in the registry of health related problems and diseases, would result in improved data quality and comparability, which would in turn lead to better‐informed policies, improved patient outcomes, and more effective resource allocation.

A similar Delphi‐based approach has been successfully used by our team to develop an MDS for cow's milk protein allergy in children [19]. The present study extends this methodology to the field of OCD.

Several measurement tools have already been developed for gathering data on OCD, including: The Yale‐Brown Obsessive Compulsive Scale (Y‐BOCS), which is a clinician‐rated scale designed to assess the severity of OCD symptoms [20]. The Obsessive Compulsive Inventory‐Revised (OCI‐R), which is a self‐report questionnaire designed to measure the severity of OCD symptoms [21] and the Dimensional Obsessive Compulsive Scale (DOCS), which is a self‐report questionnaire that measures the severity of OCD symptoms and related distress [22]. The Children's Yale‐Brown Obsessive Compulsive Scale (CY‐BOCS), is a clinician‐rated scale designed to assess OCD symptoms in children and adolescents. Each of these tools has its own strengths and limitations. While existing data collection tools primarily focus on assessing symptom severity, this designed MDS aims to fill the gaps by incorporating additional data dimensions. Including administrative, demographic, and multidimensional data will provide a more comprehensive understanding of OCD, enabling better‐informed care and more targeted research.

## 2 Materials and Methods

### 2.1 Study Design and Setting

This descriptive cross‐sectional study was conducted in the following steps.

#### 2.1.1 Step 1: Extracting the Data Elements Required to Design the MDS for OCD

##### 2.1.1.1 Search Process

To begin, a literature review was initiated on July 10, 2024. In the first step to retrieve related resources (articles, reports, forms and theses), scientific databases such as the Web of Science, PubMed, Scopus, Google Scholar, SID, Magiran and Google search engine were reviewed by keywords including “mental health,” “mental disorders,” “obsessive‐compulsive disorder,” “OCD,” “minimum data set,” “MDS,” and “data set.” The inclusion criteria were articles, reports, forms and theses in Persian and English languages, published from 2000 to 2024. Letters to the editor, short communications, articles with no abstract and full text, articles that focused on other aspects of OCD (irrelevant to the present study), articles that did not provide clear information were excluded.

Data were extracted from the related retrieved resources and entered into a data extraction form which was validated by two health information management experts and included fields for data elements and references were then reviewed by two psychiatrists affiliated with Kerman Medical Sciences University.

#### 2.1.2 Step 2: Final Agreement on Data Elements via Delphi Technique

##### 2.1.2.1 Study Participation and Sampling

The study population included specialists and subspecialists from fields of [1] Psychology [2] psychiatry [3] epidemiology and [4] health information management working in educational and medical centers associated with Kerman University of Medical Sciences.

As is typically the case in Delphi‐based research, the number of experts is generally between 15 and 20. Accordingly, for this study, 20 specialists and subspecialists in the aforementioned fields were selected to participate. Their demographic information is displayed in Table 1.

**Table 1:** Demographic information of experts.

Type of medical specialties	Frequency	Sex	Education degree	Average work experience
Psychologist	7	Male: 2	Specialist	24 years
		Female: 5		
Psychiatrist	8	Male: 5	Specialist	22 years
		Female: 3		
Epidemiologist	2	Male: 1	Specialist	13 years
		Female: 1		
Health information manager	3	Male: 0	Specialist	10 years
		Female: 3		

The following inclusion criteria were used to choose participants:

- Employment of participants in medical and educational centers associated with Kerman University of Medical Sciences.
- Having at least 5 years of experience in diagnosing and treating OCD (for Psychologists and Psychiatrists).
- Having experience in designing measurement development studies for diseases or chronic conditions (for Health Information Managers and Epidemiologists).

##### 2.1.2.2 Data Gathering Tool and Method

Based on the data elements collected in the previous step, a questionnaire was designed. The questionnaire consisted of two sections [1] demographic information of the experts and [2] the data elements required to design the MDS for OCD.

The researcher categorized the data elements into seven groups [1] Administrative data [2] Clinical presentation [3] Clinical history [4] Treatment history [5] Physical health [6] Environmental and sociocultural factors that may be contributing to OCD symptoms, and [7] Service utilization. To ensure the accuracy of these categories, two sessions were held with two psychiatrists and one psychologist affiliated with Kerman University of Medical Sciences. In the first session, the specialists reviewed the collected data and in the second session, they shared their opinions on categorizing the data elements, and suggested additional elements if needed.

We used a standard 5‐point Likert scale (from “completely agree” to “completely disagree”). The validity of the questionnaire was confirmed by two health information managers and two psychiatrists and based on their opinions; some changes were made. Then the agreed questionnaire was designed.

The Delphi technique was implemented by distributing the questionnaire in person to expert participants on September 10, 2024. All questionnaires were completed and collected by September 20, and the data was then inputted into SPSS software version 23 for analysis. The frequency of choices was calculated as percentages. Data elements that received approval from more than 75% of the experts were accepted, while those receiving approval between 50% and 75% were re‐evaluated. Data elements that received approval from less than 50% of the experts were removed from the analysis [23].

In the second round of the Delphi technique, a new questionnaire was developed based on the analysis of the first round. This questionnaire included data elements that had received approval ratings between 50% and 75%. After a month following the first round, the questionnaire was distributed to the same group of experts. Their responses were collected and analyzed to further refine the data elements under consideration.

We assessed reliability using Cronbach's alpha (α = 0.85 in SPSS v23), indicating excellent internal consistency.

### 2.2 Reporting Guideline

The reporting of this modified Delphi study has been guided by the CREDES (Guidance on Conducting and REporting DElphi Studies) framework [24]. A completed CREDES checklist is provided as Appendix 1 to ensure transparency and completeness of reporting.

**Figure 1:**
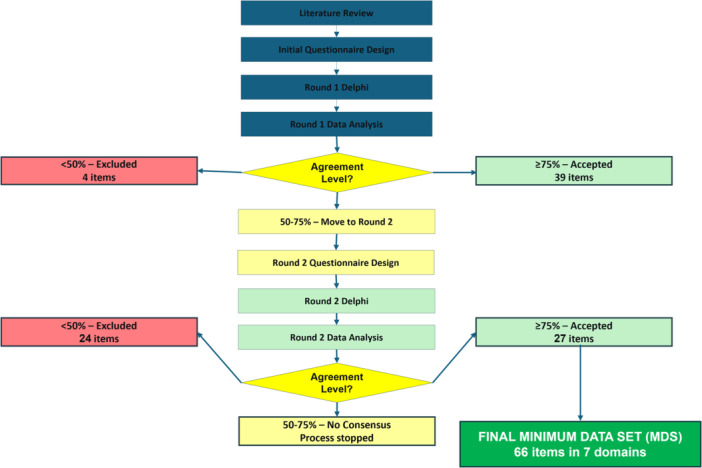
Flowchart of the modified Delphi process. The figure illustrates the sequential steps of the study, from the initial literature review (94 candidate items) through two Delphi rounds to the final 66‐item Minimum Data Set (MDS). Consensus was defined as ≥ 75% agreement. Items with 50%–75% agreement proceeded to Round 2, while items with < 50% agreement were excluded. The process was stopped after Round 2 by panel consensus.

The stages of the modified Delphi process are illustrated in Figure 1.

## 3 Results

As shown in Tables 2 and 3, this modified Delphi study achieved consensus on 66 essential data elements for OCD assessment through a two‐round evaluation process with expert panelists. The results demonstrate strong agreement on clinical measures while identifying areas requiring further consideration in demographic and service utilization domains.

**Table 2:** Delphi consensus results for OCD diagnostic scale data elements: agreement percentages and retention status.

Category	Subcategory	Data element	The agreement percentage in the initial round of Delphi		The result of the initial round of Delphi	The agreement percentage in the second next of Delphi	The result of the next round of Delphi
1. Administrative data	I. Demographic data	1. Patient ID	60%		*	70%	√
		2. Type of health insurance	55%		*	50%	×
		3. Name	50%		*	45%	×
		4. Last name	50%		*	40%	×
		5. National code	40%		*	45%	×
		6. Father's name	60%		*	50%	×
		7. Date of birth	80%		√		
		8. Age	75%		*	80%	√
		9. Blood Group	60%		*	50%	×
		10. Ethnicity or race	75%		*	80%	√
		11. Gender	85%		√		
		12. Marital status (single, married, widowed, divorced)	90%		√		
		13. Education level ◦ ≤ 12 years (non‐tertiary) ◦ > 12 years (tertiary education)	80%		√		
		14. Socioeconomic status (SES) ◦ Low: Unskilled laborers, unemployed ◦ Middle: Skilled workers, clerical ◦ High: Professionals, executives	80%		√		
		15. Health insurance coverage	50%		*	50%	×
	II. Geographic History	1. Current residence: (with name of the place) ◦ Urben ◦ Rural	65%		*	75%	√
		2. Duration of current residence	60%		*	50%	×
		3. Previous residences	50%		*	50%	×
		4. Birthplace	75%		*	80%	√
	III. Family and Socioeconomic status	1. Highest education household (< High School/College).	70%		*	75%	√
		2. parents SES ◦ Low: Unskilled laborers, unemployed ◦ Middle: Skilled workers, clerical ◦ High: Professionals, executives	75%		*	75%	√
		3. Number of children in the family	75%		*	85%	√
		4. Patient birth order	80%		√		
		5. Contact number of one of the parents or a close family member	65%		*	50%	×
	IV. Legal and Ethical consideration	1. Consent for treatment and data collection	75%		*	75%	√
		2. Consent for sharing information with other healthcare providers or family members	75%		*	80%	√
		3. Consent for sharing information for research purposes	75%		*	75%	√
		4. Consent for follow‐up contact: ◦ Yes, I agree to be contacted [email/secure app/phone number]. ◦ No	75%		*	75%	√
		5. Legal guardianship (if applicable)	45%		×		
2. Clinical presentation	I. OCD symptoms and severity (Based on DY‐BOCS)	Obsessions: (Check all that apply): ◦ Contamination (fear of germs/illness) ◦ Harm (aggressive/violent thoughts) ◦ Symmetry/Exactness (“just right” obsessions) ◦ Forbidden/Taboo (sexual/religious thoughts) ◦ Hoarding‐Related (If hoarding is OCD‐driven) ◦ Other: _____________ (free text) *Severity*: Mild (1) ‐ Extreme (5) scale for each selected (clinician‐administered)	100%		√		
		Compulsions: (Check all that apply): ◦ Washing/Cleaning ◦ Checking ◦ Repeating ◦ Ordering/Arranging ◦ Mental Rituals ◦ Reassurance‐Seeking ◦ Other: _____________ *Time:* Spent (Required for each checked) *t*: < 1 h/day | 1–3 hrs | > 3 hrs	100%		√		
		Insight Level (DSM‐5 Specifier): ◦ Good/Fair (“My fears are excessive”) ◦ Poor (“My fears are realistic”)	100%		√		
	Functional impairment (Based on WHODAS 2.0 Domains (Gold standard))	◦ Cognition ◦ Self‐Care ◦ Work/School ◦ Social Scoring: 0 (None) to 4 (Extreme), (clinician‐rated)	90%		√		
	II. Comorbid conditions	Mental Health: ◦ Depression (PHQ*‐9 ≥ 10) ◦ GAD (GAD*‐7 ≥ 10) ◦ Tic Disorder (YGTSS* if available) ◦ ADHD (ASRS*‐5) ◦ Suicidality (PHQ‐9 Item 9 ≥ 1) ◦ Other: _______________	100%		√		
		Physical Health (OCD‐relevant only): ◦ PANDAS/PANS (if pediatric) ◦ Autoimmune (e.g., Hashimoto's) ◦ Neurological (e.g., Tourette's) ◦ Other: _____________	95%		√		
3. Clinical history	I. Pregnancy and birth factors	1. Maternal Age at Delivery (years) ◦ < 20 ◦ 20–35 ◦ > 35	80%		√		
		2. Delivery Mode (Vaginal Delivery or Cesarean Delivery)	50%		*	50%	×
		3. Preterm Birth (< 37 weeks)	70%		*	75%	√
		4. Multiple births	70%		*	65%	√
		5. Prolonged labor	55%		*	50%	×
		6. Fetal hypoxia at birth	60%		*	50%	×
		7. Low 5‐min Apgar Score (less than 7)	65%		*	75%	√
	II. Age of OCD onset	1. Age of OCD onset ◦ < 10 ◦ 10–18 ◦ > 18	100%		√		
	III. Family history of mental illness	1. Type of mental diagnosed illness (based on DSM‐5 or ICD‐11)	100%		√		
		2. Degree of certainty ◦ Confirmed diagnosis ◦ Suspected by patient	75%		*	80%	√
		3. Relation to patient	100%		√		
		4. Age of onset: ◦ < 10 ◦ 10–18 ◦ > 18	90%		√		
		5. Treatment history: types of treatment received and their effectiveness	55%		*	50%	×
	VI. Hospitalization history of the patient	1. Number of psychiatric hospitalizations #____	75%		*	75%	√
		2. Reason for admission	75%		*	85%	√
		3. Length of stay	55%		*	40%	×
		4. Complications or outcomes	50%		*	40%	×
	V. suicidality	1. Lifetime suicide attempts (past month): #____ ◦ None ◦ Passive ◦ Active	100%		√		
		2. Current suicidal ideation (PHQ‐9 Item 9 ≥ 1)	100%		√		
		3. Non‐suicidal self‐injury (NSSI)	100%		√		
		4. Safety planning: any safety plans or coping strategies that the patient has developed to prevent self‐harm behaviors	40%		×		
4. Treatment history	I. Drug Therapy	1. Medication Class ◦ SSRIs (e.g., fluoxetine, sertraline) ◦ SNRIs (e.g., venlafaxine) ◦ Clomipramine ◦ Augmentation agents (e.g., antipsychotics)	100%		√		
		2. Treatment Response (based on *Clinical Global Impression‐Improvement [CGI‐I])* *score: 1–7*	90%		√		
		3. Adequate trial (≥ 12 weeks at max tolerated dose): ◦ Yes ◦ No	90%		√		
	II. Psychotherapy	1. Cognitive Behavioral Therapy (CBT) *Number of sessions completed: #____“*(≥ 12 sessions is benchmark). *“Response:* □ *Full* □ *Partial* □ *None”* (per Y‐BOCS reduction).	95%		√		
		2. Exposure and Response Prevention (ERP) *Number of sessions completed: #____“*(≥ 12 sessions is benchmark). *“Response:* □ *Full* □ *Partial* □ *None”* (per Y‐BOCS reduction).	90%		√		
		3. Mindfulness‐Based Therapy	50%		*	45%	×
		4. Psychodynamic Therapy	60%		*	40%	×
		5. Other type of Psychotherapy	80%		√		
	III. Alternative treatment	1. Complementary/Alternative Medicine (CAM) ◦ Herbal (e.g., inositol) ◦ Acupuncture ◦ Other: ______	80%		√		
		2. Reason for use ◦ Lack of conventional treatment efficacy ◦ Preference ◦ Side effects	70%		*	80%	√
	3. Treatment adherence and preferences	1. Missed ≥ 30% of medication doses in past month: □ Yes □ No	80%		√		
		2. Completed ≥ 80% of ERP/CBT sessions: □ Yes □ No	85%		√		
		3. Reasons for non‐adherence: ◦ side effects ◦ cost ◦ stigma	70%		*	70%	√
		4. Strategies to improve adherence: any strategies that may help the patient adhere to their treatment plan	30%		×		
		5. Preferred modality: ◦ Medication ◦ Therapy ◦ Combined ◦ CAM	80%		*	80%	√
		6. Treatment setting: the patient's preferred setting for treatment, such as outpatient or inpatient	55%		*	45%	×
		7. Treatment provider: the patient's preferred type of treatment provider, such as a psychiatrist or a psychologist	50%		*	45%	×
5. Physical health		1. Sleep disturbances linked to obsessions: Do obsessions/compulsions delay bedtime or disrupt sleep?” □ Yes □ No	80%		√		
		2. Insomnia severity (based on Insomnia Severity Index [ISI])	80%		√		
		3. Sleep apnea	50%		*	50%	×
		4. Eating disorders	55%		*	50%	×
		5. Sedentary behavior	75%		*	75%	√
		6. Any physical OCD‐Comorbid Conditions: ◦ Autoimmune/metabolic ◦ Neurological	80%		√		
6. Environmental and Sociocultural factors that may be contributing to OCD symptoms	1. Family and caregiver support	1. family members participation: Do family members participate in rituals or provide reassurance? (Use the Family Accommodation Scale (FAS)) ◦ Never ◦ Sometimes ◦ Frequently	70%		*	80%	√
		2. family routines disruptions: How often does the patient's OCD disrupt family routines? ◦ Rarely ◦ Weekly ◦ Daily	45%		×		
	2. Life events	1. Trauma History: Any history of abuse (physical/sexual/emotional)? □ Yes □ No	85%		√		
		2. Did OCD symptoms worsen after trauma?” □ Yes □ No	90%		√		
	3. Stressors	1. Recent Stressors (past 12 months): ◦ Checklist ◦ Job loss ◦ Bereavement ◦ Major relocation	80%		√		
	4. Cultural and societal factors	1. Stigma	85%		√		
		2. Religious/Spiritual OCD	80%		√		
		3. Loneliness	80%		√		
		4. Discrimination	75%		*	75%	√
	5. Coping strategies	1. Maladaptive Coping: *Do you use rituals to reduce anxiety?”* □ Yes □ No	70%		*	75%	√
		2. Adaptive Coping: *Have you tried ERP techniques independently?”* □ Yes □ No	75%		*	75%	√
7. Service utilization	Mental health services	1. Received ERP from an OCD‐trained provider? □ Yes □ No	85%		√		
		2. “Trials of ≥2 SSRIs at adequate dose/duration? □ Yes □ No (Defines treatment resistance)	60%		*	50%	×
		3. Telehealth Utilization: *“Used teletherapy for OCD?* □ Yes □ No	80%		√		
		4. Substance Use Services? □ Yes □ No	75%		*	75%	√
		5. Emergency Services? □ Yes □ No	50%		*	45%	×

*Note:* √: Final acceptance, ×: Final exclusion and *: resurvey in the next round of Delphi. *PHR: Patient Health Questioner, * GAD: Generalized Anxiety disorder, * YGTSS: Yale Global Tic Severity scale, * ASRS: Adult ADHD Self‐report Scale.

**Table 3:** Final retained data elements after Delphi consensus rounds.

Category	Subcategory	Data element
Administrative data	Demographic data	Patient ID
		Age
		Ethnicity or race
		Gender
		Marital status (single, married, widowed, divorced)
		Education level ◦ ≤ 12 years (non‐tertiary) ◦ > 12 years (tertiary education)
		Socioeconomic status (SES) ◦ Low: Unskilled laborers, unemployed ◦ Middle: Skilled workers, clerical ◦ High: Professionals, executives
	Geographic History	Current residence ◦ Urben ◦ rural
		Birthplace
	Family and Socioeconomic status	Highest education household (< High School/College).
		parents SES ◦ Low: Unskilled laborers, unemployed ◦ Middle: Skilled workers, clerical ◦ High: Professionals, executives
		Number of children in the family
		Patient birth order
	Legal and Ethical consideration	Consent for treatment and data collection
		Consent for sharing information with other healthcare providers or family members
		Consent for sharing information for research purposes
		Consent for follow‐up contact: ◦ Yes, I agree to be contacted [email/secure app/phone number]. ◦ No
8. Clinical presentation	III. OCD symptoms and severity (Based on DY‐BOCS)	Obsessions: (Check all that apply): ◦ Contamination (fear of germs/illness) ◦ Harm (aggressive/violent thoughts) ◦ Symmetry/Exactness (“just right” obsessions) ◦ Forbidden/Taboo (sexual/religious thoughts) ◦ Hoarding‐Related (If hoarding is OCD‐driven) ◦ Other: _____________ (free text) *Severity*: Mild (1) ‐ Extreme (5) scale for each selected(clinician‐administered)
		Compulsions: (Check all that apply): ◦ Washing/Cleaning ◦ Checking ◦ Repeating ◦ Ordering/Arranging ◦ Mental Rituals ◦ Reassurance‐Seeking ◦ Other: _____________ *Time:* Spent (Required for each checked) *t*: < 1 h/day | 1–3 hrs | > 3 hrs
		Insight Level (DSM‐5 Specifier): ◦ Good/Fair (“My fears are excessive”) ◦ Poor (“My fears are realistic”)
	Functional impairment (Based on WHODAS 2.0 Domains (Gold standard))	◦ Cognition ◦ Self‐Care ◦ Work/School ◦ Social Scoring: 0 (None) to 4 (Extreme), (clinician‐rated)
	IV. Comorbid conditions	Mental Health: ◦ Depression (PHQ*‐9 ≥ 10) ◦ GAD (GAD*‐7 ≥ 10) ◦ Tic Disorder (YGTSS* if available) ◦ ADHD (ASRS*‐5) ◦ Suicidality (PHQ‐9 Item 9 ≥ 1) ◦ Other: _______________
		Physical Health (OCD‐relevant only): ◦ PANDAS/PANS (if pediatric) ◦ Autoimmune (e.g., Hashimoto's) ◦ Neurological (e.g., Tourette's) ◦ Other: _____________
Clinical history	Pregnancy and birth factors	Maternal Age at Delivery (years) ◦ < 20 ◦ 20–35 ◦ > 35
		Preterm Birth (< 37 weeks)
		Multiple births
		Low 5‐min Apgar Score (less than 7)
	Age of OCD onset	Age of OCD onset ◦ < 10 ◦ 10–18 ◦ > 18
	Family history of mental illness	Type of mental diagnosed illness (based on DSM‐5 or ICD‐11)
		Degree of certainty ◦ Confirmed diagnosis ◦ Suspected by patient
		Relation to patient
		Age of onset: ◦ < 10 ◦ 10–18 ◦ > 18
	Hospitalization history of the patient	Number of psychiatric hospitalizations #____
		Reason for admission
	suicidality	Lifetime suicide attempts (past month): #____ ◦ None ◦ Passive ◦ Active
		Current suicidal ideation (PHQ‐9 Item 9 ≥ 1)
		Non‐suicidal self‐injury (NSSI)
Treatment history	Drug Therapy	Medication Class ◦ SSRIs (e.g., fluoxetine, sertraline) ◦ SNRIs (e.g., venlafaxine) ◦ Clomipramine ◦ Augmentation agents (e.g., antipsychotics)
		Treatment Response (based on Clinical Global Impression‐Improvement [CGI‐I]) Score: 1–7
		Adequate trial (≥ 12 weeks at max tolerated dose): ◦ Yes ◦ No
	Psychotherapy	Cognitive Behavioral Therapy (CBT) Number of sessions completed: #____“(≥ 12 sessions is benchmark). “Response: □ Full □ Partial □ None” (per Y‐BOCS reduction).
		Exposure and Response Prevention (ERP) Number of sessions completed: #____“(≥ 12 sessions is benchmark). “Response: □ Full □ Partial □ None” (per Y‐BOCS reduction).
		Other type of Psychotherapy
	Alternative treatment	Complementary/Alternative Medicine (CAM) ◦ Herbal (e.g., inositol) ◦ Acupuncture ◦ Other: ______
		Reason for use ◦ Lack of conventional treatment efficacy ◦ Preference ◦ Side effects
	Treatment adherence and preferences	Missed ≥ 30% of medication doses in past month: □ Yes □ No
		Completed ≥ 80% of ERP/CBT sessions: □ Yes □ No
		Reasons for non‐adherence: ◦ Side effects ◦ Cost ◦ Stigma
		Preferred modality: ◦ Medication ◦ Therapy ◦ Combined ◦ CAM
Physical health		Sleep disturbances linked to obsessions: Do obsessions/compulsions delay bedtime or disrupt sleep?” □ Yes □ No
		Insomnia severity (based on Insomnia Severity Index [ISI])
		Sedentary behavior
		Any physical OCD‐Comorbid Conditions: ◦ Autoimmune/metabolic ◦ Neurological
Environmental and Sociocultural factors that may be contributing to OCD symptoms	Family and caregiver support	Do family members participate in rituals or provide reassurance? (Use the Family Accommodation Scale (FAS)) ◦ Never ◦ Sometimes ◦ Frequently
	Life events	Trauma History: Any history of abuse (physical/sexual/emotional)? □ Yes □ No
		Did OCD symptoms worsen after trauma?” □ Yes □ No
	Stressors	2. Recent Stressors (past 12 months): ◦ Checklist ◦ Job loss ◦ Bereavement ◦ Major relocation
	Cultural and societal factors	Stigma
		Religious/Spiritual OCD
		Loneliness
		Discrimination
	Coping strategies	3. Maladaptive Coping: Do you use rituals to reduce anxiety?” □ Yes □ No
		Adaptive Coping: Have you tried ERP techniques independently?” □ Yes □ No
Service utilization	Mental health services	Received ERP from an OCD‐trained provider? □ Yes □ No
		Telehealth Utilization: “Used teletherapy for OCD? □ Yes □ No
		Substance Use Services? □ Yes □ No

### 3.1 Round 1 Outcomes

In the initial evaluation of 94 candidate items, panelists reached immediate consensus on 39 items, predominantly in clinical domains. The OCD symptom and severity assessment items based on DY‐BOCS showed perfect (100%) agreement, including:

- Obsession and compulsion subtypes
- Time spent on rituals
- Insight level assessment

Comorbidity assessment for both mental health and physical health conditions also achieved 95%–100% approval.

51 items requiring further consideration progressed to Round 2, while 4 items (legal guardianship, Safety planning, Strategies to improve adherence and family routines disruptions) were immediately excluded due to low agreement. The demographic category proved most contentious, with only 6 of 15 items achieving immediate consensus, primarily basic identifiers like age, gender, and marital status.

### 3.2 Round 2 Outcomes

The second evaluation yielded consensus on 27 additional items, bringing the final accepted total to 66. Key patterns emerged:

1. Clinical History Items
  ◦ Pregnancy/birth factors showed mixed results, with preterm birth, multiple births and Apgar scores gaining acceptance, while delivery mode, Prolonged labor and Fetal hypoxia at birth, remained excluded
  ◦ Family history items maintained strong agreement (80‐100%), except treatment history details (excluded at 50%)
2. Treatment Components:
  ◦ Medication‐related items achieved 90%–100% consensus
  ◦ While CBT and ERP measures were overwhelmingly accepted (90%–95%), alternative therapies like mindfulness were excluded (45%)
3. Environmental Factors:
  ◦ Cultural considerations (stigma, religious OCD) showed strong agreement (75%–85%)
  ◦ Family accommodation measures required Round 2 evaluation but ultimately gained approval (80%)

### 3.3 Final Composition

The approved MDS comprises seven domains:

1. Administrative Data (17 items)
2. Clinical Presentation (6 items)
3. Clinical History (14 items)
4. Treatment History (12 items)
5. Physical Health (4 items)
6. Environmental Factors (10 items)
7. Service Utilization (3 items)

### 3.4 Notable Exclusions

28 items failed to reach consensus, primarily:

- Detailed demographic identifiers (name, national code)
- Specific service utilization metrics (emergency service use)
- Less common treatment modalities (psychodynamic therapy)
- Some birth history details (delivery mode, prolonged labor)

Notably, among perinatal factors, only those with stronger empirical support (e.g., preterm birth, Apgar scores) were retained, while items with weaker or inconclusive evidence (e.g., delivery mode, prolonged labor, fetal hypoxia at birth) were excluded from the final MDS due to lack of expert consensus (agreement < 50%). Regarding service utilization, although its importance was acknowledged, items such as emergency service use did not reach consensus primarily due to feasibility concerns raised by the expert panel, particularly regarding inconsistent documentation in low‐resource settings.

### 3.5 Consensus Patterns

The results reveal three distinct tiers of expert agreement:

1. Strong Consensus (90%–100%): Clinical symptom measures, core comorbidities, and treatment fundamentals
2. Moderate Consensus (70%–85%): Developmental history and environmental factors
3. Low Consensus (< 70%): Specific demographic details and service utilization metrics

The study achieved its primary objective of identifying essential data elements while highlighting areas where clinical experts prioritize symptom‐focused measures over administrative details. The final 66‐item MDS provides a balanced framework capturing the multidimensional nature of OCD while remaining feasible for clinical implementation.

## 4 Discussion

Existing OCD assessment tools, such as the Y‐BOCS and DOCS, primarily focus on symptom severity [20, 22]. As discussed in the introduction, our proposed multidimensional MDS extends this approach by integrating administrative, demographic, clinical, and sociocultural data, thereby offering a more comprehensive foundation for epidemiological research, public health policy, and personalized care.

The proposed MDS is designed to complement, not replace, existing OCD assessment tools such as the Y‐BOCS, DOCS, and OCI‐R [21], While these instruments focus primarily on symptom severity and dimensional assessment of obsessive‐compulsive symptoms, our MDS captures a broader range of data domains—including administrative, demographic, environmental, service utilization, and developmental history—that are not covered by these tools. There is minimal direct overlap between MDS items and existing symptom scales, as the MDS intentionally excludes detailed symptom severity ratings (e.g., time spent on rituals, distress levels, interference). Where some conceptual overlap exists (e.g., family history items), the MDS provides more detailed and structured data collection than is typically captured in routine clinical assessment. Therefore, we envision the MDS being used alongside validated symptom measures to provide a more comprehensive understanding of each patient's context.

The inclusion of administrative and demographic data in this MDS allows for more comprehensive epidemiological studies. Researchers can examine OCD prevalence across different populations, identify potential risk factors, and inform public health policies targeting specific demographic groups. Demographic data are also essential for understanding vital events such as fertility, mortality, morbidity, and migration [14]. Furthermore, documenting disparities in access to care, quality of treatment, and clinical outcomes can guide targeted interventions to improve healthcare services [25]. By addressing limitations noted in previous research, this MDS enables a deeper understanding of factors influencing quality of life and functional impairment in OCD patients. Beyond epidemiological applications, this MDS facilitates personalized treatment planning by providing a more complete understanding of patients' backgrounds, including age, gender, education, and occupation. Olatunji et al. demonstrated that understanding individual patient characteristics helps guide the selection of optimal therapeutic interventions [26], and our MDS offers a holistic view of patient contexts to enable clinicians to better customize treatment plans and improve outcomes. Finally, by emphasizing administrative and demographic factors alongside clinical symptoms, this MDS addresses knowledge gaps in the literature and allows researchers to investigate the relationships between demographic factors and treatment outcomes, ultimately leading to more effective interventions [27].

This MDS acknowledges the potential impact of pregnancy and birth factors on OCD as they may contribute to the development and understanding of the disorder. A large population‐based birth cohort study of 2.4 million children in Sweden demonstrated that several perinatal factors, including preterm birth and low Apgar distress scores, are associated with a higher risk of developing OCD [28].

The “Family History of Mental Illness” category plays a vital role in OCD research and clinical practice. A family history of OCD and related disorders increases an individual's risk for developing OCD and may influence treatment outcomes. A recent systematic review and meta‐analysis of genetic epidemiology confirmed that OCD is a highly familial disorder, with a phenotypic heritability of approximately 50%. The study found that familial recurrence rates are particularly elevated among first‐degree relatives of children and adolescent probands, and that the higher symptom correlations in monozygotic twins are primarily due to additive genetic effects and non‐shared environmental components [29]. Therefore, capturing family history helps clinicians develop personalized treatment plans and investigate genetic and environmental contributors to OCD.

The “Environmental and Sociocultural Factors” category in our MDS addresses the need for a comprehensive understanding of OCD beyond clinical symptoms. Research has shown that various external factors, such as family dynamics and cultural influences, can affect the development, expression, and severity of OCD [30]. A recent systematic review by Baldini et al. (2024) demonstrated a significant association between childhood trauma (especially emotional abuse/neglect) and more severe OCD symptoms in adulthood [31]. By incorporating this category into our MDS, we enable healthcare professionals and researchers to examine these external factors' impact on OCD symptoms and treatment outcomes. Understanding the role of environmental and sociocultural factors in OCD management allows for the development of tailored interventions that address patients' unique needs [32, 33, 34].

The inclusion of the “Service Utilization” category in our MDS sheds light on how OCD patients access and engage with healthcare services. By understanding the types of services utilized, frequency of use, and any gaps in service provision, researchers and healthcare providers can identify potential barriers to care and develop targeted interventions to improve patient access to appropriate treatment [35]. In a study by Williams et al. (2012), researchers investigated the obstacles that hinder African Americans with OCD from accessing treatment. The study found that concerns about cost were significantly higher for individuals without insurance compared to those with public or private insurance plans. By evaluating various barriers across different countries, policymakers can make informed decisions on resource allocation to improve treatment accessibility for OCD patients [36].

Current OCD data collection tools, like the Yale‐Brown Obsessive‐Compulsive Scale (Y‐BOCS) and the Dimensional Obsessive‐Compulsive Scale (DOCS), mainly concentrate on evaluating symptom severity and specific obsessive‐compulsive dimensions. These instruments might not adequately address other significant factors such as service utilization, environmental and sociocultural influences, and physical health. In contrast, our proposed MDS adopts a more comprehensive perspective by integrating these critical components of OCD management. By including categories like “Service Utilization,” “Environmental and Sociocultural Factors,” and “Physical Health,” our MDS provides a more comprehensive understanding of OCD and its management. This broader approach supports the development of tailored interventions and targeted public health policies to enhance patient outcomes.

This proposed MDS serves as a valuable addition to existing OCD assessment tools by providing a broader perspective on the disorder and encouraging a more integrated approach to care. By incorporating administrative, demographic, and clinical data, this MDS offers a comprehensive understanding of OCD management, enabling tailored treatment plans and focused interventions to improve patient outcomes, as emphasized by Olatunji et al. (2013) in their study on the importance of considering various aspects of OCD for effective management [26].

### 4.1 Clinical and Research Applications

The proposed MDS is designed for both clinical and research applications. In clinical practice, it complements existing tools like the Y‐BOCS by capturing broader contextual data (demographic, environmental, service utilization) to inform personalized care, while the Y‐BOCS remains the standard for symptom severity monitoring. For routine use, we anticipate collecting a core subset of 25–30 items during initial intake. In research and registry settings, the full 66‐item MDS can serve as a standardized framework for establishing regional or national OCD registries, enabling data comparability across sites. A flexible “core + optional modules” approach allows adaptation to both resource‐constrained and comprehensive research settings.

### 4.2 Future Directions for Validation and Implementation

While the Delphi consensus established content validity for the proposed OCD‐MDS, several next steps are necessary before widespread implementation.

1. Piloting and Feasibility Assessment
  We plan to conduct a pilot study in two clinical settings in Kerman, Iran, enrolling 100 patients with confirmed OCD diagnosis over a 3‐month period. Feasibility will be assessed using the following metrics: completion rate (percentage of patients with complete MDS data), time burden (average time required for data collection), missing data rate (percentage of missing items per patient), and user acceptability (semi‐structured interviews with clinicians and data collectors). Based on pilot results, the MDS will be refined before larger‐scale implementation.
2. Validity and Reliability Assessment
  A comprehensive validation study will be conducted to assess multiple psychometric properties:
  - **Test‐retest reliability:** A subset of 50 patients will complete the MDS twice, 2 weeks apart. An Intraclass Correlation Coefficient (ICC) of ≥ 0.75 will indicate excellent test‐retest reliability.
  - **Inter‐rater reliability:** Two independent data collectors will complete the MDS for 30 patients. Cohen's kappa (for categorical items) and ICC (for continuous items) will be calculated, with target values of κ ≥ 0.70 and ICC ≥ 0.75.
  - **Concurrent validity:** MDS severity measures will be compared with gold‐standard instruments including the Y‐BOCS, with a target correlation of r ≥ 0.70.
  - **Construct validity:** Convergent and discriminant validity will be assessed using hypothesis testing (e.g., strong correlation with related constructs, weak correlation with unrelated constructs).

3. Cross‐Cultural Applicability
  To ensure the MDS is useful beyond its original Iranian context, we will follow the standard WHO forward‐backward translation protocol for cross‐cultural adaptation. We plan to collaborate with researchers in at least two other countries (e.g., Turkey for a similar cultural context, and a European country for a different cultural context) to assess item relevance, comprehensibility, and measurement equivalence. A **“core + optional modules” framework** will be developed, consisting of a universally applicable core module (approximately 40‐45 items) and culture‐specific optional modules that can be added or removed based on local needs (e.g., stigma‐related items, family structure variables, religious OCD measures).

4. Registry and Clinical Integration
  We will map MDS items to international data standards such as the OMOP Common Data Model and SNOMED CT to facilitate interoperability with existing registries. We also plan to integrate the MDS into the mental health patient registry at Kerman University of Medical Sciences and will seek collaboration with the International OCD Foundation (IOCDF) for broader dissemination.

## 5 Limitations

This study has several limitations. The expert panel was limited to a single institution in Iran, which restricts the generalizability of the proposed MDS to other cultural and geographical contexts where OCD presentation and healthcare systems may differ. Additionally, the absence of patient representatives is another limitation. These issues will be addressed in our planned cross‐cultural validation studies, as outlined in the “Future Directions” section.

Future research should evaluate the MDS's impact on patient outcomes and public health policy, with ongoing cross‐cultural validation to support its global applicability.

## 6 Conclusion

This study developed an MDS for OCD that can serve as a foundation for establishing related patient registries. In countries like Iran, where accurate statistics on OCD prevalence and contributing factors are lacking, utilizing such registries can significantly enhance the diagnosis, management, and control of OCD. These registries can provide valuable insights for researchers, healthcare professionals, policymakers, and affected individuals, ultimately improving the quality of life for patients and their families. Moreover, increasing public awareness through such initiatives can lead to earlier diagnosis and better symptom management, potentially reducing disease complications and overall burden.

Unlike existing symptom‐focused tools [20, 21, 22], our MDS integrates essential factors such as service utilization, environmental and sociocultural influences, and physical health.

As we continue to strive for more effective OCD management strategies, the importance of a multidimensional approach becomes increasingly apparent. By integrating various data sources, our MDS not only complements existing assessment tools but also facilitates personalized care and a deeper understanding of OCD within diverse populations. In doing so, this MDS has the potential to transform OCD management, ensuring that patients receive the most comprehensive and tailored care possible.

## Author Contributions

**Farzaneh Asadilari:** conceptualization, methodology, software, data curation, supervision, resources, formal analysis, project administration, visualization, investigation, writing – original draft, writing – review and editing. **Ahmad Naghibzadeh‐Tahami:** conceptualization, methodology, investigation, supervision, resources, writing – original draft, writing – review and editing, visualization. **Sadrieh Hajesmaeel‐Gohari:** conceptualization, investigation, writing – original draft, writing – review and editing, visualization, methodology, supervision, resources. **Mohammad Mehdi Ghaemi:** conceptualization, methodology, validation, writing – review and editing.

## Funding

The authors have nothing to report.

## Ethics Statement

The study was conducted under the auspices of Kerman University of Medical Sciences, with ethical approval (IR.KMU.REC.1403.500).

## Conflicts of Interest

The authors declare no conflicts of interest.

## Transparency Statement

F.A.L. affirms that this manuscript is an honest, accurate, and transparent account of the study being reported; that no important aspects of the study have been omitted; and that any discrepancies from the study as planned (if relevant) have been explained.

## Supporting information


Supporting File

## Data Availability

The data that support the findings of this study are available from the corresponding author upon reasonable request.
