# Supplementary material for: A Multidimensional Data Set for Obsessive‐Compulsive Disorder to Strengthen Research and Care: A Cross‐Sectional Delphi Study
Source: Health Sci Rep. 2026 Aug 13;9(8):e72861. doi: 10.1002/hsr2.72861 (PMC13473749; doi:10.1002/hsr2.72861)
Supplement: Supplementary file 1 [file HSR2-9-e72861-s001.docx]

**Appendix 1: CREDES Checklist for Reporting Delphi Studies(1)**

**Manuscript Title:** A Multidimensional Data Set for Obsessive-Compulsive Disorder to Strengthen Research and Care: A Cross‑Sectional Delphi Study

| **Item** | **CREDES Recommendation** | **Location in Manuscript** | **Reported** |
| --- | --- | --- | --- |
| **1. Purpose and rationale** | The purpose of the study is clearly defined and demonstrates the appropriateness of using the Delphi technique as a method to achieve the research aim. A rationale for choosing the Delphi technique as the most suitable method is provided. | Introduction (pages 3-4) | ✅ |
| **2. Expert panel** | Criteria for expert selection and transparent information on recruitment of the expert panel are reported. This includes sociodemographic details, information on expertise, and (non)response rates over the ongoing iterations. | Methods: Study participation and sampling (pages 5-6); Table 3 | ✅ |
| **3. Description of the methods** | The methods employed are comprehensible. This includes information on preparatory steps (e.g., how available evidence was synthesized), piloting of survey instruments, design of the survey instrument(s), number and design of survey rounds, methods of data analysis, and processing and synthesis of responses. | Methods: Search process, Data gathering tool and method, Delphi technique (pages 4-6) | ✅ |
| **4. Procedure** | A flow chart illustrates the stages of the Delphi process, including the preparatory phase, the actual "Delphi rounds", interim steps of data processing and analysis, and concluding steps. | Figure 1 (page 7) | ✅ |
| **5. Definition and attainment of consensus** | It is comprehensible to the reader how consensus was achieved throughout the process, including strategies to deal with non‑consensus (e.g., items with 50-75% agreement proceeded to Round 2; items with <50% agreement were excluded). | Methods: Delphi technique (page 4-6) | ✅ |
| **6. Results** | Results for each round are reported separately to make the evolution of consensus transparent. This includes figures showing average group responses, changes between rounds, and any modifications of the survey instrument. | Results: Round 1 Outcomes, Round 2 Outcomes (pages7-9) | ✅ |
| **7. Discussion of limitations** | The reporting includes a critical reflection on potential limitations and their impact on the resulting guidance. | Discussion: Limitations (pages 9-13) | ✅ |
| **8. Adequacy of conclusions** | The conclusions adequately reflect the outcomes of the Delphi study with a view to the scope and applicability of the resulting practice guidance. | Conclusion (page 13) | ✅ |
| **9. Publication and dissemination** | The resulting guidance (the Minimum Data Set) is clearly identifiable in the publication, including recommendations for translation into practice and implementation. | Results: Final Composition (pages7-9); Discussion: Clinical and Research Applications, Future Directions for Validation and Implementation (pages 10-12) | ✅ |

1.	Jünger S, Payne SA, Brine J, Radbruch L, Brearley SG. Guidance on Conducting and REporting DElphi Studies (CREDES) in palliative care: Recommendations based on a methodological systematic review. Palliat Med. 2017;31(8):684-706.
